# Phase Behavior of Amorphous/Semicrystalline Conjugated Polymer Blends

**DOI:** 10.3390/polym12081726

**Published:** 2020-07-31

**Authors:** Gada Muleta Fanta, Pawel Jarka, Urszula Szeluga, Tomasz Tański, Jung Yong Kim

**Affiliations:** 1 Institute of Engineering Materials and Biomaterials, Faculty of Mechanical Engineering, Silesian University of Technology, 44-100 Gliwice, Poland; gadamuleta2019@gmail.com (G.M.F.); Pawel.Jarka@polsl.pl (P.J.); 2 School of Materials Science and Engineering, Jimma Institute of Technology, Jimma University, Post Office Box 378 Jimma, Ethiopia; 3 Centre of Polymer and Carbon Materials, Polish Academy of Sciences, M. Curie-Skłodowska 34 Street, 41-819 Zabrze, Poland; uszeluga@cmpw-pan.edu.pl; 4 School of Chemical Engineering, Jimma Institute of Technology, Jimma University, Post Office Box 378 Jimma, Ethiopia

**Keywords:** phase behavior, conjugated polymer, polymer blend, all-polymer solar cells, melting point depression, amorphous, semicrystalline, polymer thermodynamics

## Abstract

We report the phase behavior of amorphous/semicrystalline conjugated polymer blends composed of low bandgap poly[2,6-(4,4-bis(2-ethylhexyl)-4H-cyclopenta [2,1-b;3,4-b′]dithiophene) -*alt*-4,7(2,1,3-benzothiadiazole)] (PCPDTBT) and poly{(*N*,*N*′-bis(2-octyldodecyl)naphthalene -1,4,5,8-bis(dicarboximide)-2,6-diyl)-*alt*-5,5′-(2,2′-bithiophene)} (P(NDI2OD-T2)). As usual in polymer blends, these two polymers are immiscible because Δ*S_m_* ≈ 0 and Δ*H_m_* > 0, leading to Δ*G_m_* > 0, in which Δ*S_m_*, Δ*H_m_*, and Δ*G_m_* are the entropy, enthalpy, and Gibbs free energy of mixing, respectively. Specifically, the Flory–Huggins interaction parameter (*χ*) for the PCPDTBT /P(NDI2OD-T2) blend was estimated to be 1.26 at 298.15 K, indicating that the blend was immiscible. When thermally analyzed, the melting and crystallization point depression was observed with increasing PCPDTBT amounts in the blends. In the same vein, the X-ray diffraction (XRD) patterns showed that the π-π interactions in P(NDI2OD-T2) lamellae were diminished if PCPDTBT was incorporated into the blends. Finally, the correlation of the solid-liquid phase transition and structural information for the blend system may provide insight for understanding other amorphous/semicrystalline conjugated polymers used as active layers in all-polymer solar cells, although the specific morphology of a film is largely affected by nonequilibrium kinetics.

## 1. Introduction

Conjugated polymer blends composed of polymer donor (P_D_) and polymer acceptor (P_A_) have been used as an active material for all-polymer solar cells (all-PSCs), leading to a high power conversion efficiency (PCE) of ~10–14.4% [1,2,3,4,5,6,7,8]. Here, when P_D_ and P_A_ are mixed together, the goal of this mixing is not to make a miscible blend unlike the versatile commercial blends, e.g., poly(vinyl chloride) (PVC)-butadiene/acrylonitrile copolymer (NBR) and polystyrene-poly(2,6-dimehtyl -1,4-phenylene oxide) (PPO) blends [9], but an immiscible (or partially miscible) blend for the separation of Frenkel excitons at the P_D_/P_A_ heterojunction [10,11]. In general, the ideal size of phase-separated domains is ~10–20 nm because the exciton has a limited diffusion length (*L_D_* = ~10 nm) due to low dielectric constants of organic macromolecules [12,13,14]. However, if two homopolymers are mixed, the polymer blends may undergo macrophase separation because of the increased Gibbs free energy of mixing [15]. On the other hand, when P_D_ and P_A_ are linearly linked by covalent bonds, these block copolymers may undergo microphase separation into ordered structures, such as cubic sphere, hexagonal cylinder, bicontinuous gyroid, and lamellae [16,17]. Hence, considering the necessity of nanoscale phase separation (much smaller than micro-/macro-scale domains) for organic photovoltaics (OPV), we need to understand the polymer–polymer thermodynamic behavior in the P_D_/P_A_ blend films.

If P_D_ and P_A_ are amorphous, there are two types of phase separation in liquid–liquid (L–L) phase transition: nucleation and growth (NG), and spinodal decomposition (SD) [18,19]. Here, NG proceeds in the metastable region, whereas SD takes place in the unstable region without any energy barrier. However, if P_D_ and/or P_A_ are semicrystalline, the liquid–solid (L–S) phase transition (crystallization) also takes place with L–L phase transition [20,21,22]. Indeed, in many P_A_/P_D_ blend systems, they contain a stereoregular (or regioregular) polymer, leading to both L–L and L–S phase transition, directly affecting the morphologies of P_D_/P_A_ blend films. Importantly, the pre-formed aggregation through L–S phase transition in OPV blend solutions may play a significant role in generating nanoscale phase domains instead of macrophase separation [20].

In previous studies, we reported the order-disorder phase equilibria of regioregular poly(3-hexylthiophene-2,5-dyil) (r-reg P3HT) solution [20]. Then, we also studied the phase diagrams of low bandgap poly[2,6-(4,4-bis(2-ethylhexyl)-4H–cyclopenta[2,1-b;3,4-b′]dithiophene) -*alt*-4,7(2,1,3-benzothiadiazole)] (PCPDTBT) solution as a function of solvent, polymer, and chain length [21]. Recently, we investigated the phase behavior of crystalline/crystalline conjugated polymer blends, composed of r-reg P3HT and poly{(*N*,*N*′-bis(2-octyldodecyl)naphthalene-1,4,5,8 -bis(dicarboximide)-2,6-diyl)-*alt*-5,5′-(2,2′-bithiophene)} (P(NDI2OD-T2)). Then, we also explained the upper critical solution temperature (UCST) behavior of each polymer solution as a function of solvent species, for which the Flory–Huggins lattice theory was employed [22].

In this study, we further extended our works to an amorphous/crystalline conjugated polymer blend, for which PCPDTBT and P(NDI2OD-T2) were chosen as a model system. Here, PCPDTBT is a p-type polymer acting as P_D_ [23,24,25,26]. Interestingly, if PCPDTBT with bulky branched alkyl side chains (ethyl hexyl groups) has a relatively low number average molecular weight ($M_{n}$ ≈ 3.2 kg/mol), it shows amorphous or marginally crystallizable behavior, indicating that the chain ends (just like impurities) may disturb polymer crystallization process [21]. However, PCPDTBT with high $M_{n}$ ≈ 35 kg/mol could be highly crystallized when this polymer was processed with exposure to chlorobenzene (CB) vapors and/or with some solvent additives such as 1,8-diiodooctane (DIO), 1,8-octanedithiol (ODT), and 1,8-dichlorooctane (DCO) [23,24,25,26]. Note that in such a case, we may not rule out the effect of substrate on polymer’s crystallization. On the other hand, P(NDI2OD-T2) is a highly crystallizable n-type polymer, acting as P_A_ in all-PSCs or n-channels, exhibiting very high electron mobility ($\mu_{n}$ ≈ 0.85 cm^2^/Vs) in the top-gate bottom-contact (TGBC) organic thin-film transistor (OTFT) [27,28,29,30,31,32,33]. Here, it is notable that P(NDI2OD-T2)’s glass transition temperature (*T_g_*) was reported to be −70 °C and −40 °C, based on dynamic mechanical analysis (DMA) and differential scanning calorimetry (DSC), respectively [34,35]. Hence, inspired by the important properties of these two polymers [21,23,24,25,26,27,28,29,30,31,32,33,34,35], we further studied the thermodynamic behavior of PCPDTBT/P(NDI2OD-T2) blends based on the thermal and structural analyses. Specifically, the melting phenomena of crystalline P(NDI2OD-T2) with added amorphous PCPDTBT material was investigated using DSC and confirmed by X-ray diffraction (XRD), in which π-stacking was observed to be dependent on the amount of amorphous PCPDTBT in the blend film. Finally, the morphologies of the pure polymer and 1:1 blend films were investigated using the tapping-mode atomic force microscopy (AFM).

## 2. Materials and Methods

### 2.1. Materials

P(NDI2OD-T2) [*M_n_* = 32.1 kg/mol, *M_w_* = 90.0 kg/mol, polydispersity index (PDI) = 2.8, and molecular formula = (C_62_H_88_N_2_O_4_S_2_)_n_] was purchased from 1-Material, Inc. (Quebec, Canada). PCPDTBT [*M_n_* = 4.50 kg/mol, *M_w_* = 13.5 kg/mol, PDI = 3.0, and molecular formula = (C_31_H_38_N_2_S_3_)_n_], chlorobenzene (CB), and chloroform (CF) were provided from Sigma-Aldrich, Inc. (Taufkirchen, Germany). All these materials were used as received without further purification.

### 2.2. Film Processing

Two conjugated polymers, PCPDTBT and P(NDI2OD-T2), were dissolved in the mixed solvents, CB:CF = 1:1 (wt. ratio) according to literature reports [36,37,38]. The total concentration of the blend system was 10 mg/mL. After the solvent dissolved completely, the samples were spin-coated on a glass substrate with the dimensions of 1.5 × 1.5 cm^2^ for thin film deposition. Prior to spin-coating, the glass substrate was sequentially cleaned in deionized water, chloroform, and isopropanol for 5 min, respectively, and then dried under a nitrogen atmosphere. The condition of spin-coating was 2000 rpm for 15 s in the air, leading to the film thickness of about 140 nm. The annealing condition for a film was 180 °C for 15 min.

### 2.3. Thermal Property Characterization

Thermal analysis was performed using DSC (2920-DSC, TA Instruments, Champaign, IL, USA) to characterize the transition temperature of polymers at the scan rate of 10 °C/min under N_2_. Thermal decomposition of polymers was monitored using thermogravimetric analysis (TGA; a METTLER TOLEDO STARe System, Warsaw, Poland), in which samples were heated from 50 to 600 °C using a conventional heating ramp with a scan rate of 10 °C/min under N_2_.

### 2.4. Film Characterization

X-ray diffraction (XRD) measurements (PANalytical Inc., Malvern, United Kingdom; PX 3040 PR; Cu Kα radiation; *λ* = 1.5418 Å) were performed to examine the structure and ordering of a film on glass substrate at room temperature. According to the XRD’s instrumental set-up condition, the diffraction angle (2θ) ranged from 10° to 100°. The morphologies of the polymer films were characterized by the tapping-mode AFM (XE-100 Park Systems, Mannheim, Germany). Here, the data were analyzed using the software Park Systems XEI.

## 3. Results

**Figure 1:**
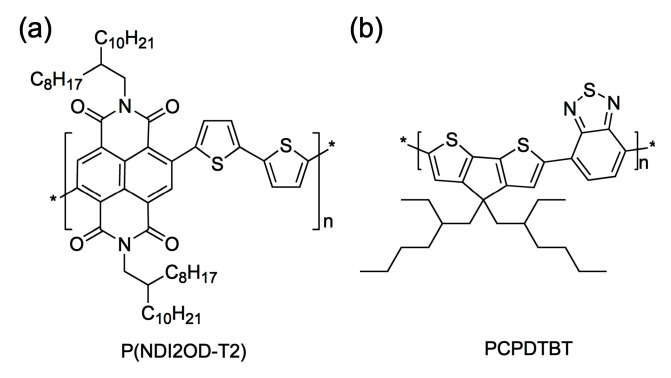
Chemical structures of (**a**) P(NDI2OD-T2) (poly{(*N*,*N*′-bis(2-octyldodecyl)naphthalene -1,4,5,8-bis(dicarboximide)-2,6-diyl)-*alt*-5,5′-(2,2′-bithiophene)}) and (**b**) PCPDTBT (poly[2,6-(4,4-bis(2-ethylhexyl)-4H-cyclopenta [2,1-b;3,4-b′]dithiophene) -*alt*-4,7(2,1,3-benzothiadiazole)]), respectively.

Figure 1 shows the chemical structures of (a) P(NDI2OD-T2) and (b) PCPDTBT. In the previous works, we reported that the solubility parameter (*δ*) of P(NDI2OD-T2) is 7.99 [22], whereas that of PCPDTBT (*M_n_* = 3.2 kg/mol and PDI = 2.2) is 10.70 [21]. Note that in this work, PCPDTBT has *M_n_* = 4.5 kg/mol and PDI = 3.0, indicating a very minor difference between the two PCPDTBT samples. Hence, based on the former *δ* data, the Flory–Huggins interaction parameter ($\chi_{ij}$) [22,39,40] for the PCPDTBT/P(NDI2OD-T2) polymer blends could be estimated using Equation (1),

(1) $$\chi_{ij}=\frac{{\hat{V}}_{s}}{RT}{\left(\delta_{i}-\delta_{j}\right)}^{2}$$

where ${\hat{V}}_{s}$ (= lattice site volume) and $\delta_{i\;or\;j}$ were the molar volume of a solvent [${\hat{V}}_{s}$ = (112.56 g/mol)/(1.11 g/cm^3^) = 101.41 cm^3^/mol for CB] and the solubility parameter [subscript *i* or *j* = 1, 2; PCPDTBT = 1 and P(NDI2OD-T2) = 2]. *R* and *T* were the gas constant and temperature (K). Then $\chi_{12}$ was estimated to be 374.82 K/T, indicating that $\chi_{12}=1.26$ at *T* = 298.15 K and $\chi_{12}$ = 0.5 at *T* = 749.64 K. Hence, considering the critical value of interaction parameter [41], ${\left(\chi_{12}\right)}_{crit}=\frac{1}{2}\cdot {\left(\frac{1}{\sqrt{r_{1}}}+\frac{1}{\sqrt{r_{2}}}\right)}^{2}$ ≈ 0.022 and $\chi_{12}>{\left(\chi_{12}\right)}_{crit}$, we conclude that PCPDTBT and P(NDI2OD-T2) were immiscible as usual for many polymer–polymer mixtures. Here, we used $r_{1}=\frac{\left(\frac{M_{n,1}}{\rho_{1}}\right)}{\left(\frac{MW_{s}}{\rho_{s}}\right)}=\frac{\left(\frac{4500}{1}\right)}{\left(\frac{112.56}{1.11}\right)}\approx 44$ and $r_{2}=\frac{\left(\frac{M_{n,2}}{\rho_{2}}\right)}{\left(\frac{MW_{s}}{\rho_{s}}\right)}=\frac{\left(\frac{32100}{1.1}\right)}{\left(\frac{112.56}{1.11}\right)}\approx 288$, in which $r_{1}\;\mathrm{and}\;r_{2}$ were the relative molar volumes of component 1 and 2, $M_{n,1}\;\mathrm{and}\;M_{n,2}$ were the number average molecular weights of component 1 and 2, $MW_{s}$ was the molecular weight of a solvent (CB), and $\rho_{1},\;\rho_{2},\;\mathrm{and}\;\rho_{s}$ were the densities of component 1, 2, and solvent, respectively. At this moment, keep in mind that the Flory–Huggins theory should be understood qualitatively, not quantitatively.

As demonstrated in the aforementioned example, the PCPDTBT/P(NDI2OD-T2) mixture was an immiscible blend, which could be understood based on $\Delta G_{m}=\Delta H_{m}-T\Delta S_{m}$ where $\Delta G_{m}$ was the Gibbs free energy of mixing, $\Delta H_{m}$ was the enthalpy of mixing, and $\Delta S_{m}$ was the entropy of mixing. When mixing long chain macromolecules, $\Delta S_{m}\approx 0$ and $\Delta H_{m}>0$ leading to $\Delta G_{m}>0$. Note that the stability condition for a single phase is ${\left({\frac{\partial^{2}\Delta G_{m}}{\partial \phi }}_{i}{}^{2}\right)}_{T,P}>0$ where $\phi_{i}$ is volume fraction of component *i* and *P* is pressure. Hence, most polymer–polymer blends including PCPDTBT and P(NDI2OD-T2) are immiscible but phase-separated. Usually, the same structural units are more attractive to each other than different ones, leading to aggregation and clusters, i.e., the phase separation of blends.

**Figure 2:**
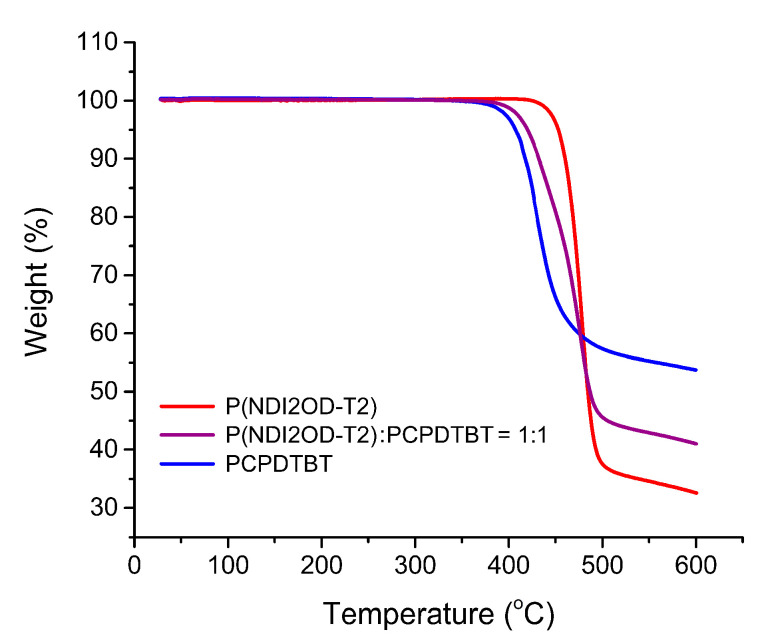
TGA curves for P(NDI2OD-T2), P(NDI2OD-T2)/PCPDTBT blend, and PCPDTBT.

**Figure 3:**
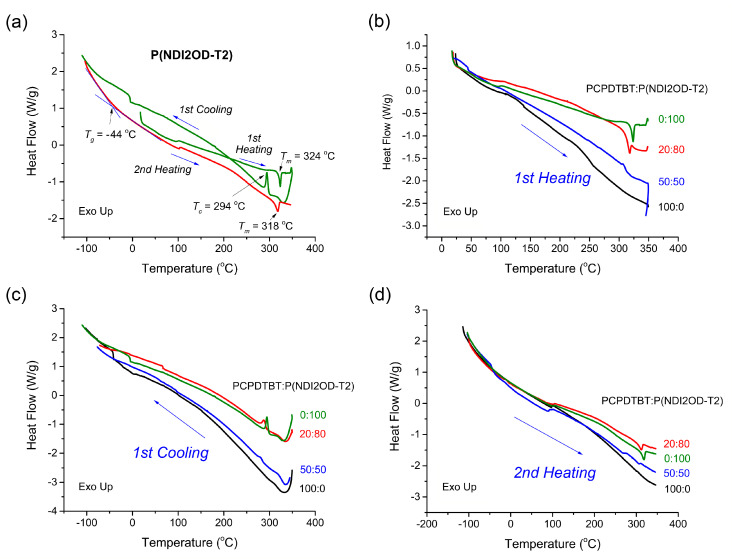
DSC curves. (**a**) Heating-cooling-heating curves for P(NDI2OD-T2). (**b**) 1st heating, (**c**) 1st cooling, and (**d**) 2nd heating curves for P(NDI2OD-T2):PCPDTBT = 100:0, 80:20, 50:50 and 0:100 (wt. ratio), respectively.

Figure 2 exhibits TGA data, indicating that PCPDTBT and P(NDI2OD-T2) generally decompose at around ~400–500 °C in organic molecules. Figure 3 shows the DSC thermograms for the PCPDTBT/P(NDI2OD-T2) blends. As shown in Figure 3a, P(NDI2OD-T2) was a semicrystalline polymer with *T_m_* = 324 °C (1st heating) or 318 °C (2nd heating) and *T_c_* = 294 °C (1st cooling). On the other hand, PCPDTBT did not show any melting point, indicating it was a typical amorphous polymer [42]; see Figure 3b–d. About the glass transition temperature (*T_g_*), P(NDI2OD-T2) showed it at −44 °C in the DSC thermogram, which agreed with Gu et al.’s report (*T_g_* = −40 °C) within experimental uncertainties [35]. Although in the previous work [21], PCPDTBT showed *T_g_* ≈ 112 °C, in this work we could not observe it clearly. That is because, as shown in Figure 3d, some artifacts were observed for all the samples around 100 °C from the DSC instrumental conditions. Note that *T_g_* is a second order transition, but in many conjugated polymer systems, it is hard to determine *T_g_* due to a weak signal in DSC thermal curves. It may be from the rigid or semirigid backbone structure of conjugated polymers, leading to relatively small free volume (and heat capacity) changes around the second-order transition when compared to flexible coil polymers.

**Figure 4:**
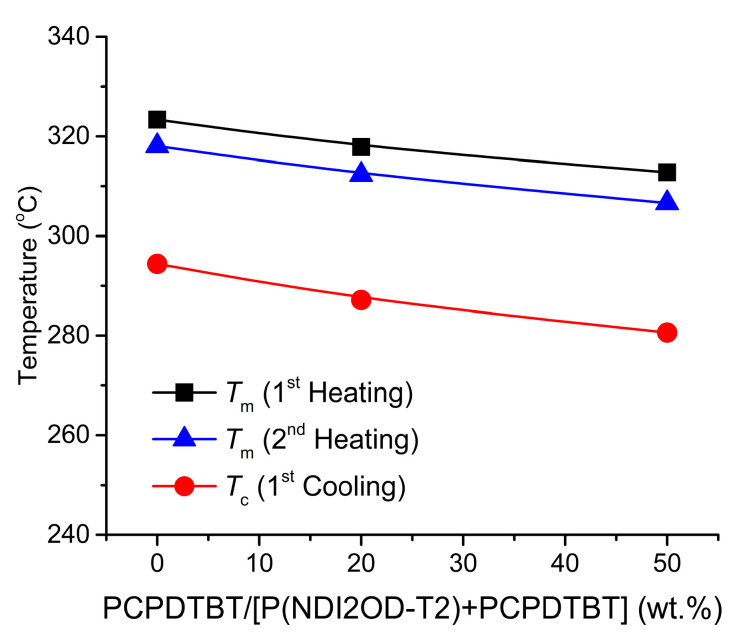
Melting and crystallization point depression of P(NDI2OD-T2)/PCPDTBT blends.

Note that, in this work, our research interest lies primarily in the S-L phase equilibria (the 1st order transition) of conjugated polymer blends composed of the semicrystalline P(NDI2OD-T2) and the amorphous PCPDTBT polymers because it is relatively more clear than other 2nd order transition. As shown in Figure 3a, the 1st heating curve, the melting point is depressed from 323.35 °C at 100% P(NDI2OD-T2) to 317.83 °C at 80% to 312.79 °C at 50%, which is a trend observed in the 1st cooling and 2nd heating curves, as well. Here, note that in Figure 3b, at PCPDTBT:P(NDI2OD-T2) = 50:50, the melting peak looks very broad, indicating that, in a blend system, a semicrystalline component should be required at least at 50% for observing a clear and sharp *T_m_* peak. Then, the results were summarized in Figure 4, displaying both the *T_m_* and *T_c_* depression, in which the average difference (Δ*T = T_m_ − T_c_*) between *T_m_* and *T_c_* is 30.0 ± 1.6 °C in the first-cycling thermal curve. The origin of this depression comes from the new equilibrium between crystalline lamellar and amorphous (liquid) chains of P(NDI2OD-T2) when a diluent PCPDTBT was introduced into the semicrystalline P(NDI2OD-T2) system for forming polymer blends. Specifically, PCPDTBT has one order lower molecule weight, *M_n_* = 4.5 kg/mol compared to P(NDI2OD-T2) with *M_n_* = 32.1 kg/mol, indicating that many available end groups in PCPDTBT may serve as impurities in blend systems. However, a liquid state PCPDTBT chain molecule itself may act as impurities in semicrystalline P(NDI2OD-T2) blends, depressing the melting point of the polymer blend systems.

**Figure 5:**
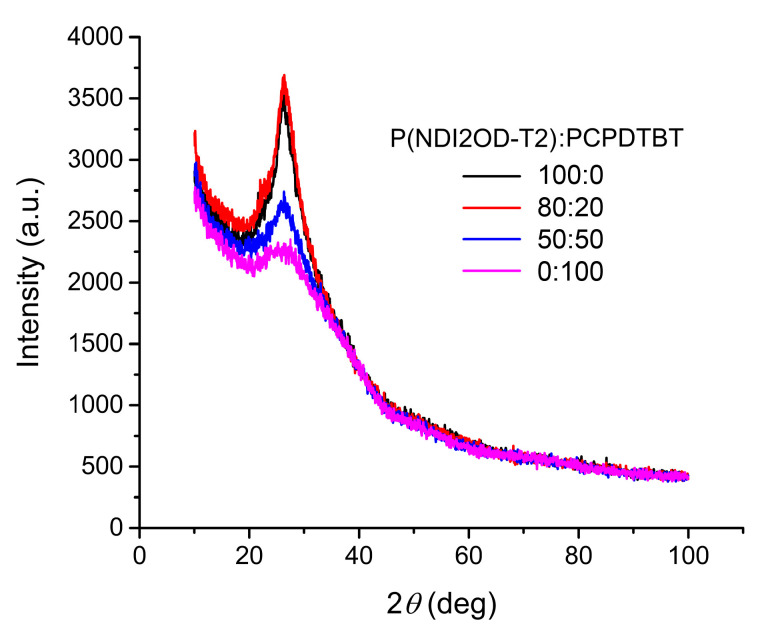
XRD patterns of P(NDI2OD-T2):PCPDTBT blends with 100:0, 80:20, 50:50 and 0:100 (wt. ratio).

Figure 5 shows the XRD patterns of pure P(NDI2OD-T2), mixed P(NDI2OD-T2)/PCPDTBT blends, and pure PCPDTBT, respectively, in the range of 2θ = 10–100°. At 2θ = ~26°, some peaks related with π-π stacking are observed. Here, the crystallite size (*t*) could be estimated based on Scherrer’s equation, t=0.9λ/(B cos θ) [43,44], where λ is the X-ray wavelength (= 0.154 nm) and *B* is the full width at half maximum (FWHM) at the diffraction angle θ. The results are summarized in Table 1, indicating that the crystallite size decreases from 2.5 nm at 100% P(NDI2OD-T2) to 2.0 nm at 50%. In the case of PCPDTBT, its nominal crystallite size is very small ~ 1.4 nm, indicating that it is an amorphous material because the length scale is around the unit cell. Importantly, if we recall the DSC results in Figure 3, when the crystallite size (related with π-stacking) is larger than 2 nm, we may observe the melting transition in DSC. It is notable also that, in polymer science, the typical unit cell, chain-folded lamella and spherulite have dimensions of ~0.2–2 nm, ~10–50 nm thick and several microns wide, and ~100–1000 μm, respectively [42]. In the case of conjugated polymer, thin-film samples for OPV, we usually observe up to a lamella scale because a film thickness is ~100–200 nm. However, there are some exceptions reporting a three-dimensional spherulite structure [26,45,46,47]. At this moment, for interested readers, the detailed XRD data about P(NDI2OD-T2) films are available in the literatures [28,29,30,31,32,33], in which edge-on and face-on morphologies were also reported.

**Figure 6:**
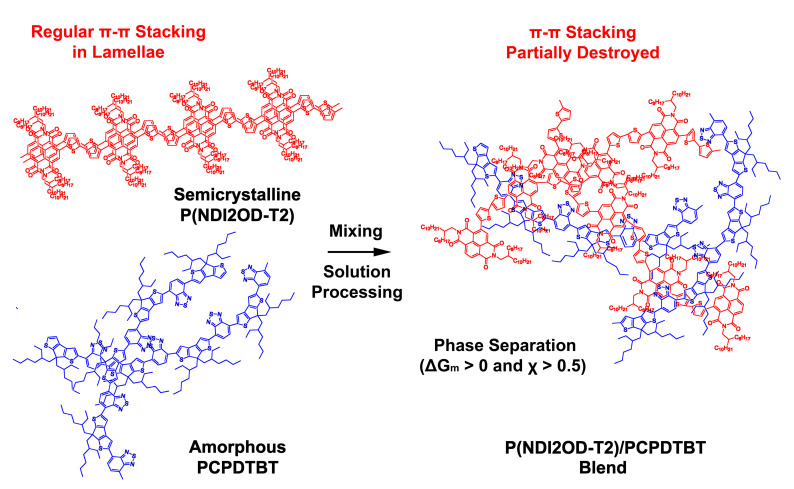
Schematic explanation of when semicrystalline P(NDI2OD-T2) and amorphous PCPDTBT polymers are mixed together through solution processing. Here, the regular π-π stacking could be destroyed by adding PCPDTBT into a crystalline P(NDI2OD-T2) lamellae.

Based on the crystallite size analysis in Table 1, as well as the melting point depression in Figure 4, we suggest the scheme displayed in Figure 6. When we add the amorphous chain molecule PCPDTBT into the semicrystalline P(NDI2OD-T2) sample, the crystalline region of P(NDI2OD-T2) may be destroyed, which could be confirmed through the decrease in XRD peaks’ intensity at 2θ = ~26°, i.e., π-π stacking, as well as the diminished melting peak in DSC thermograms in Figure 3. However, it is notable that the aforementioned phenomena do not necessarily mean the miscibility of the two polymers, i.e., they are still in phase-separate state. They are immiscible because of the thermodynamic reason, $\Delta G_{m}>0$. However, the above observation may affect the nano- and micro-structure of blend films, which should be important in all-PSCs because of the limited diffusion length of Frenkel excitons in the organic/polymer semiconductors with low dielectric constants.

**Figure 7:**
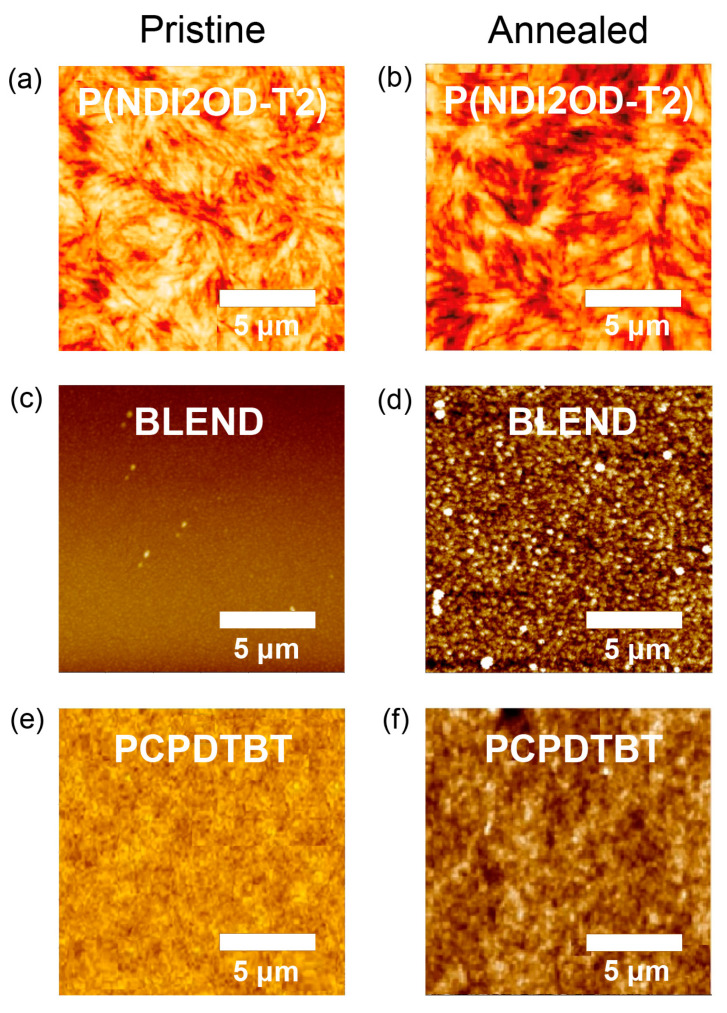
Tapping-mode AFM images of (**a**) pristine P(NDI2OD-T2), (**b**) annealed P(NDI2OD-T2), (**c**) pristine P(NDI2OD-T2):PCPDTBT = 1:1 blend, (**d**) annealed P(NDI2OD-T2):PCPDTBT = 1:1 blend, (**e**) pristine PCPDTBT, and (**f**) annealed PCPDTBT films, respectively.

Figure 7 shows the AFM images of pure P(NDI2OD-T2), P(NDI2OD-T2)/PCPDTBT blend, and pure PCPDTBT. As shown in Figure 7a, P(NDI2OD-T2) film displays rod-shaped crystalline lamellae with a few μm. However, when P(NDI2OD-T2) was mixed with PCPDTBT with a 1:1 wt. ratio, the morphology changed dramatically. Here, elongated crystal domains were destroyed and diminished into a granular shape. Finally, PCPDTBT exhibits somewhat homogenous images when compared with P(NDI2OD-T2). This smoothness is expected because PCPDTBT is amorphous (disordered liquid or glass) based on both the DSC and XRD results.

## 4. Conclusions and Future Works

Two low bandgap conjugated polymers, semicrystalline P(NDI2OD-T2) and amorphous PCPDTBT, were mixed together for a study on phase behavior. Here, P(NDI2OD-T2) is a high-performance n-type semiconductor whereas PCPDTBT is a p-type one when applied to optoelectronic devices. In this work, although two polymers were immiscible with $\chi_{12}=1.26$ at *T* = 298.15 K, the blend exhibited the melting and crystallization temperature depression phenomena because PCPDTBT acts as a diluent or impurity for the P(NDI2OD-T2) lamellae. In this process, PCPDTBT molecules make it difficult for the P(NDI2OD-T2) chains to form lamellae, leading them to destroy the regular π-π stacking in the polymer lamellae, which was confirmed through XRD and AFM data. Specifically, when the size of crystallite-related π-stacking was larger than 2 nm, we observed the melting points in DSC. Finally, when we apply conjugated polymer blends to electronic and optoelectronic devices, it is very important to understand a phase-separation mechanism leading to a specific morphology. Further study will be designed for understanding other photovoltaic polymer blends with amorphous/amorphous, semicrystalline/amorphous, and semicrystalline/semicrystalline structures.

## Figures and Tables

**Table 1:** Crystallite size (*t*) and *d*-spacing of P(NDI2OD-T2):PCPDTBT blends with 100:0, 80:20, 50:50, and 0:100 (wt. ratio) at the diffraction angle θ, when X-ray has wavelength (λ) of 0.154 nm. Herein, *d*-spacing between lattice planes is calculated based on Bragg’s law, λ=2d sin θ.

P(NDI2OD-T2):PCPDTBT	2θ (°)	θ (°)	*B* (Radians)	*t* (nm)	*d*-Spacing (nm)
100:0	26.16	13.08	0.05667	2.5	0.34
80:20	26.31	13.05	0.06319	2.3	0.34
50:50	26.26	13.13	0.06606	2.0	0.34
0:100	25.47	12.74	0.09980	1.4	0.35

## Abbreviations

B	full width at half maximum
d	distance between crystallographic planes (*d*-spacing)
$L_{D}$	diffusion length
$M_{n}$	number average molecular weight
$M_{n,1\;\mathrm{or}\;2}$	number average molecular weight of component 1 or 2
$M_{w}$	weight average molecular weight
$MW_{s}$	molecular weight of a solvent
P	pressure
$P_{A}$	polymer acceptor
$P_{D}$	polymer donor
R	the gas constant
$r_{1\;\mathrm{or}\;2}$	relative molar volume of component 1 or 2
T	temperature
$T_{c}$	crystallization temperature
$T_{g}$	glass transition temperature
$T_{m}$	melting temperature
t	crystallite size
${\hat{V}}_{s}$	molar volume of a solvent
$\Delta G_{m}$	Gibbs free energy of mixing
$\Delta H_{m}$	enthalpy of mixing
$\Delta S_{m}$	entropy of mixing
δ	solubility parameter
$\delta_{i\;\mathrm{or}\;j}$	solubility parameter of component *i* or *j*
θ	diffraction angle
λ	X-ray wavelength (= 0.154 nm)
$\mu_{n}$	electron mobility
$\phi_{i}$	volume fraction of component *i*
$\rho_{1\;\mathrm{or}\;2\;\mathrm{or}\;s}$	density of component 1 or 2 or solvent
χ	Flory–Huggins interaction parameter
$\chi_{ij}$	Flory–Huggins interaction parameter between component *i* and *j*
${\left(\chi_{12}\right)}_{crit}$	Flory–Huggins interaction parameter between component 1 and 2 at critical point
AFM	atomic force microscopy
all-PSCs	all-polymer solar cells
CB	chlorobenzene
CF	chloroform
DCO	1,8-dichlorooctane
DIO	1,8-diiodooctane
DMA	dynamic mechanical analysis
DSC	differential scanning calorimetery
FWHM	full width at half maximum
L–L	liquid–liquid
L–S	liquid–solid
NBR	butadiene/acrylonitrile copolymer
NG	nucleation and growth
ODT	1,8-octanedithiol
OPV	organic photovoltaics
OTFT	organic thin-film transistor
P(NDI2OD-T2)	poly{(*N*,*N*′-bis(2-octyldodecyl)naphthalene -1,4,5,8-bis(dicarboximide)-2,6-diyl)-*alt*-5,5′-(2,2′-bithiophene)}
PCE	power conversion efficiency
PCPDTBT	poly[2,6-(4,4-bis(2-ethylhexyl)-4H-cyclopenta [2,1-b;3,4-b′]dithiophene) -*alt*-4,7(2,1,3-benzothiadiazole)]
PDI	polydispersity index
PPO	polystyrene-poly(2,6-dimehtyl -1,4-phenylene oxide)
PVC	poly(vinyl chloride)
r-reg P3HT	regioregular poly(3-hexylthiophene-2,5-dyil)
SD	spinodal decomposition
TGA	thermogravimetric analysis
TGBC	top-gate bottom-contact
UCST	upper critical solution temperature
XRD	X-ray diffraction
